# An Open Question: Is the A_2A_ Adenosine Receptor a Novel Target for Alzheimer’s Disease Treatment?

**DOI:** 10.3389/fphar.2021.652455

**Published:** 2021-03-22

**Authors:** Stefania Merighi, Tino Emanuele Poloni, Lucia Pelloni, Silvia Pasquini, Katia Varani, Fabrizio Vincenzi, Pier Andrea Borea, Stefania Gessi

**Affiliations:** ^1^ Department of Translational Medicine and for Romagna, University of Ferrara, Ferrara, Italy; ^2^ Department of Neurology and Neuropathology, Golgi-Cenci Foundation and ASP Golgi-Redaelli, Abbiategrasso, Italy; ^3^ University of Ferrara, Ferrara, Italy

**Keywords:** A_2A_ adenosine receptor, Alzheimer’s disease, biomarker, neuroinflammation, therapeutic target

## Introduction

### Neurocognitive Disorder due to Alzheimer’s Disease: A Brief Overview

According to DSM5, the term neurocognitive disorder (NCD) emphasizes that the cause of mental deficit lies in a pathology affecting neuronal circuits. The early clinical stages of NCD (mild-NCD/MCI) are characterized by functional preservation of everyday activities. Instead, if the disorder has a functional impact it is defined as major-NCD (dementia). On the other hand, the definition of the underlying pathology allows for the etiological classification of NCD (American Psychiatric Association, 2013; Sachdev et al., 2015). Based on the pathological deposition of proteins in brain tissue, NCD due to AD is characterized by a dual proteinopathy in which neurodegeneration is associated with the deposition of amyloid and phosphorylated TAU protein (pTAU). AD is the main age-related degenerative NCD progressively involving memory, complex attention, executive functions, language, and visual-perceptual functions. Personality and behavioural changes are also frequent further complicating the clinical course. On the other hand, due to the late involvement of the movement centers, motor function is usually spared until the most advanced stages of the disease. The AD syndromic evolution reflects the progressive spread of pTAU pathology from the allocortex (entorhinal cortex and hippocampus) to the neocortex (Elahi and Miller, 2017; Hanseeuw et al., 2019). Observing the neuropathology of AD is the starting point for deciphering its pathophysiological mechanisms and, therefore, identifying the biomarkers of the disease and the possible therapeutic targets. The macroscopic pathological feature characterizing advanced AD is diffuse brain atrophy due to widespread neurodegeneration causing synaptic and neuronal loss. Actually, the disease begins decades earlier with amyloid accumulation in the neocortex but amyloid deposition, which is very common even in physiological aging, is not sufficient to cause AD. The fundamental question is: what triggers neurodegeneration? Probably, the excess of amyloid-beta (A*β*) induces neurodegeneration through toxic oligomers. Indeed, soluble A*β* oligomers cause a synaptic reduction with a decrease in long-term potentiation and memory. Moreover, oligomers can reduce blood flow in brain capillaries and induce hyperphosphorylation of the AD-relevant epitopes of TAU protein (Selkoe and Hardy 2016; Nortley et al., 2019). Thus, A*β* load triggers neurodegeneration through oligomers which induce unbalanced activation of neuronal kinases resulting in excessive production of pTAU that, in turn, aggregates in pTAU toxic oligomers and spreads from its initial location in allocortex to neocortex. Together, oligomeric A*β*, synaptic pTAU aggregates and glial inflammatory activation are the main neurotoxic factors involved in the manifestation of a clinically relevant neurocognitive disorder (Perez-Nievas et al., 2013; Jack et al., 2018a). Typically, AD pathology shows extracellular accumulation of A*β* peptides (A*β* or senile plaques), as well as the hyperphosphorylated tau protein aggregates inside the dying neurons named neurofibrillary tangles (NFT) and neuropil threads (NT). Their combination constitutes the neuritic plaque (NP), which is the most typical feature of AD neuropathology. Thus, the neuropathological definition of AD requires a combination of scores for Amyloid, TAU (Braak stages), and NP (CERAD), which constitute the ABC criteria for the grading of AD related pathology (Mirra et al., 1991; Braak et al., 2006; Montine et al., 2012). Senile and neuritic plaques, consisting of protein and cellular debris, activate reactive and inflammatory processes by astrocytes and microglia which produce cytokines (IL-1*β*, IL-6) and NLRP3 inflammosome activation that, in turn, increase neurotoxic phenomena (Serrano-Pozo et al., 2016; Ising et al., 2019). On the basis of the neuropathological picture, several biomarkers have been developed for the *in vivo* definition of the pathology. Thus, the ATN system (Amyloid-TAU-Neurodegeneration) has been set up including 1) estimate of the amyloid load: A*β* decrease in cerebrospinal fluid (CSF) and/or A*β* cortical accumulation at amyloid-PET; 2) pTAU valuation: pTAU increase in CSF and/or pTAU cortical accumulation at TAU-PET; 3) extent of neurodegeneration: atrophic pattern at brain MRI and/or hypometabolism at FDG-PET and/or increase of total-TAU in CSF (Jack et al., 2018b; Chételat et al., 2020). These markers can allow for early diagnosis or even can identify those most at risk of developing AD in a preclinical phase (before mild-NCD) in order to implement timely therapeutic interventions (Dubois et al., 2016). Nonetheless, there is now no cure for AD and this approach poses ethical problems, as well as being invasive and expensive; therefore, an intensive search for biomarkers obtainable from peripheral blood is still in progress (Lewczuk et al., 2018; Molinuevo et al., 2018).

The early mechanisms leading to A*β* accumulation and initial generation of toxic molecules are elusive and multiple, and belong to the individual trajectory of cerebral aging linked to non-modifiable genetic factors (AD-related polymorphisms, APO-E4 allele, and pathogenic mutations in PSN-1-2 and APP genes, and Williamson et al., 2009; Vermunt et al., 2019), and to modifiable factors related to the individual’s personal history including favorable behaviors (regular physical and mental activity, high education, healthy diet, social engagement) and harmful conditions (midlife obesity, diabetes, hypertension, smoke, excessive alcohol, and hearing loss) (Lourida et al., 2019; Livingston et al., 2020). Early pathogenesis of sporadic AD is quite complex. Just as there are different forms of hepatitis that lead to cirrhosis, there are different pathophysiological paths that lead to AD. However, the *sine qua non* for the development of AD pathophysiology is the accumulation of amyloid in the cerebral cortex. Indeed, many efforts are being made to reduce the presence of amyloid in the cerebral cortex, especially through the use of costly monoclonal antibodies (e.g., phase3 trials: Aducanumab, Gantenerumab; phase2 trial: Crenezumab). Actually, amyloid reduction is only one aspect of the therapeutic approach and there is increasing attention to non-amyloid targets with 121 agents having clinical trials in course for the treatment of AD (Cummings et al., 2020). Particularly, a new challenge is the development of immunotherapies capable of blocking the toxic pTAU species (Bittar et al., 2020). The multifactorial nature of the AD would require an early, long-lasting, and multi-dimensional therapeutic approach which should be personalized and based on the patient’s clinical and biomarker characteristics (Sperling et al., 2011; Cummings et al., 2018; Hara et al., 2019). The current possible intervention strategies to improve the AD course depend on the stage of pathology and progressively include: prevention measures (healthy and active lifestyle, reduction of detrimental factors), disease modifying treatments (reduction of A*β* load and toxic oligomers, containment of TAU phosphorylation, toxic pTAU species and neuroinflammation, enhancement of neuronal resilience), symptomatic therapies (modulation of synaptic functions and improvement of synaptic efficiency, and Long and Holtzman 2019). Nonetheless, it should be taken into account that many senile cases of AD present mixed pathologies and in the extreme stages of senility it becomes unrealistic to stem neurodegeneration. In this framework, adenosine receptors, especially in the hippocampus, constitute a new and interesting therapeutic target through which it is possible to modulate and improve synaptic activity, obtaining symptomatic and perhaps disease-modifying effects.

## Role of A_2A_ Adenosine Receptors in AD: State of the Art and Discussion

Adenosine is an ubiquitous autacoid derived by ATP dephosphorylation, that modulates several responses in CNS, by activating four G-protein coupled receptors, A_1_, A_2A_, A_2B_, and A_3_ present on both neuronal and glial cells (Borea et al., 2018). This nucleoside is generated at both intra– and extracellular level following AMP dephosphorylation by 5′-nucleotidases and its extracellular concentration is regulated both from equilibrative nucleoside transporters as well as through exocytosis by neurons and astrocytes (Borea et al., 2016).

Adenosine regulates several physiological functions including sleep, cognitive performances, and memory and its main role is to regulate neuron excitatory synaptic transmission by inhibitory A_1_ receptors and synaptic plasticity via facilitatory A_2A_ receptors (Cunha, 2008; Cunha, 2016; Cieślak and Wojtczak, 2018). In particular, the A_2A_ subtype, mainly present in striatal area, has been now recognized in other cerebral regions including cortex and hippocampus, where due to its expression at presynaptic level, it affects the release of excitatory neurotransmitters, like glutamate (Cunha et al., 1994; Lopes et al., 2002; Marchi et al., 2002). As for its synaptic expression in spite of episodic evidence (Canas et al., 2018), it is still debatable if there is A_2A_ receptors expression in synapses, although it is well established that A_2A_ receptors are located in hippocampal synapses, with a density about 20–time lower than in the striatum (Lopes et al., 2004; Rebola et al., 2005). Although in healthy human brains the A_2A_ receptor may exert protective functions, by regulating other proteins as BDNF, its signaling is strongly modified in the hippocampus following aging (Rebola et al., 2003; Tebano et al., 2010; Temido-Ferreira et al., 2019; Temido-Ferreira et al., 2020). In this condition, there is a rise of A_2A_ receptor and G proteins-coupling, leading to an increase in glutamate release, mGluR5-dependent NMDA receptor overstimulation, and enhanced calcium influx responsible for synaptic alterations and memory dysfunction (Temido-Ferreira et al., 2020). These findings suggest a role for this receptor subtype in the pathogenesis of different neurocognitive disorders and specifically AD (Costenla et al., 2011; Rebola et al., 2011; Horgusluoglu-Moloch et al., 2017; Temido-Ferreira et al., 2019). Indeed, synaptic dysfunction and damage are key features in early AD (Selkoe, 2002; Coleman et al., 2004). Interestingly, A_2A_ receptor is overexpressed in both frontal cortex and hippocampus of aged and transgenic-AD animals as well as in AD patients (Lopes et al., 1999; Arendash et al., 2006; Albasanz et al., 2008; Espinosa et al., 2013; Li et al., 2015; Orr et al., 2015; Pagnussat et al., 2015; Gonçalves et al., 2019; Temido-Ferreira et al., 2020). APP/PS1 mouse model of AD amyloidosis show an upregulation and activation of A_2A_ adenosine receptors hampering long-term synaptic potentiation (LTP) in hippocampal CA3 pyramidal cells (Viana da Silva et al., 2016). Several literature data report the use of pharmacological and genetic approaches to demonstrate that A_2A_ adenosine receptors block prevents synaptic damage and cognitive impairments in animal models following A*β* exposure, suggesting that A_2A_ receptor antagonists might reduce synaptotoxicity (Dall'Igna et al., 2007; Canas et al., 2009; Orr et al., 2018). Moreover, antagonism of A_2A_ adenosine receptors in animal models of Tau pathology inhibits Tau hyperphosphorylation, hippocampal neuroinflammation, while protects spatial memory and hippocampal long-term depression (Laurent et al., 2016). Accordingly, overexpression of A_2A_ adenosine receptors, in a tauopathy mouse model, increases tau hyperphosphorylation and consequent tau-dependent memory impairments (Carvalho et al., 2019).

Adenosine, deriving from an increase of ecto-5′-nucleotidase (CD73) activity in animal model of early AD, induced memory deficits, LTP impairment and synaptic markers reduction in a CD73 or A_2A_ adenosine receptor-dependent way (Gonçalves et al., 2019). Indeed, among the early mechanisms involved in memory deterioration, synaptic dysfunction and selective synaptic degeneration stand out as one of the more robust and reproducible events. In fact, the early works on neuropathological changes associated with dementia established the loss of synaptic markers as a key process (Terry et al., 1991). More recent work showed that the loss of synapses is indeed of the earliest neuropathological changes in the brains of MCI and early AD patients, namely in the hippocampus (Scheff et al., 2007; Scheff et al., 2015). Accordingly, animal studies confirmed that synaptic dysfunction is an early event at the onset of memory perturbations (Canas et al., 2009; Viana da Silva et al., 2016; Silva et al., 2018). This justifies the proposal that AD is a synaptic-based disease (Selkoe, 2002) and that synaptic modulators may be paramount to control early AD (Coleman et al., 2004). Another crucial role for the A_2A_ adenosine receptor is its important ability to modulate glial cell functions, affecting pro-inflammatory cytokines release and neuroinflammation (Illes et al., 2020). Specifically, it plays an essential role in activated microglia, located near amyloid plaques typical of AD (Franco et al., 2020), where its upregulation is responsible for a raise of M1 microglial markers (IL-1*β*, IL-6, TNF-*α*) and its antagonism prevents hippocampal LTP impairments, as well as IL-1*β* production, paventing a regulatory function for it in reducing memory dysfunction (Colella et al., 2018; Franco et al., 2019). Several studies support a role of A_2A_ receptor as drug target in both neurons and microglia to revert memory deficit and neurodegeneration in AD (Santiago et al., 2014; Cunha, 2016). Recently, it has been reported that A_2A_ subtypes interact with NMDA receptors producing A_2A_-NMDA heteromers, mainly in microglia, characterized by bidirectional cross-antagonism, where A_2A_ receptor inhibition decreases hyperactivation of glutamatergic signalling by blocking NMDA receptor-mediated currents (Rebola et al., 2008; Mouro et al., 2018; Franco et al., 2020; Temido-Ferreira et al., 2020). In addition, it forms important complexes with CB_2_ cannabinoid receptor subtypes, presenting cross-interaction, thus modifying the pathway of each other. In this heteromer, A_2A_ receptor antagonism provides an increase in CB_2_ receptor activity suggesting, for the first time, that A_2A_ receptor block rises the neuroprotective action of endocannabinoids important for AD therapy (Franco et al., 2019). Interestingly, these receptorial complexes were enhanced in a transgenic AD mouse model (Franco et al., 2020). Although these heteromers might have a role, recently, A_2A_ receptor-mediated effects in AD-related features have been attributed to monomeric forms (Temido-Ferreira et al., 2020).

Clinical data, based on the effect of caffeine consumption in elderly, encourage the use of A_2A_ adenosine antagonists to prevent memory deficits. Indeed, caffeine is the most widely consumed psychostimulant substance, present in coffee, tea, cola, chocolate, and other foods, exerting benefical effects in dementia and AD (Eskelinen et al., 2009; Eskelinen and Kivipelto, 2010; Santos et al., 2010; Gelber et al., 2011; Liu et al., 2016; Sugiyama et al., 2016; Reyes and Cornelis, 2018; Domenici et al., 2019; Dong et al., 2020; Iranpour et al., 2020). Interestingly it has been reported that non-toxic doses/concentrations of caffeine mostly act on A_2A_ receptors in the brain (Yu et al., 2009; Lopes et al., 2019). Accordingly, antagonism of A_2A_ receptors is one of the main effects of caffeine (Jacobson et al., 2020).

Specifically, coffee consumption correlated with reduction of cognitive function, with significant effects obtained with three cups of coffee per day (Ritchie et al., 2007; van Gelder et al., 2007). Indeed, a retrospective analysis during 20 years before AD development, revealed a negative correlation between coffee intake and disease diagnosis, with lower quantity of caffeine at day assumed by patients with AD in contrast to higher amounts of caffeine in control subjects (Maia and de Mendonca, 2002). Furthermore, a prospective work evaluating the effect of coffee intake assumed every day on AD development, showed a reduction of AD risk by 31%, following 5 years examination (Lindsay et al., 2002). In addition, lower caffeine levels were observed in plasma of mild cognitive impairments patients developing later dementia, in comparison to those who did not develop the disease. Therefore, high levels of caffeine were related to the lack of dementia development in a window of 2/4 years (Cao et al., 2012). Accordingly, a high daily consumption of 3–5 cups of coffee reduced the risk of dementia and AD of 65–70 and 62–64%, respectively, in comparison to a lower assumption (Eskelinen et al., 2009). More generally, caffeine intake was associated to the absence of dementia and cerebral injuries typical of AD and to an increase of long-term memories consolidation in humans (Gelber et al., 2011; Borota et al., 2014; Favila and Kuhl, 2014).

In animal models of AD, administration of caffeine, has been associated to a reduced risk for memory decline and dysfunction, beta-amyloid production and tau hyperphosphorylation (Costa et al., 2008; Arendash et al., 2009; Canas et al., 2009; Cao et al., 2009; Eskelinen and Kivipelto, 2010; Santos et al., 2010; Laurent et al., 2014; Kaster et al., 2015; Kolahdouzan and Hamadeh, 2017). Finally, recent works support the utility of caffeine intake as antioxidant and antiinflammatory agent (Janitschke et al., 2019; Sinyor et al., 2020). However, it has to be remarqued that although caffeine is an abundant bioactive molecule in coffee beverages, these have over 2,000 other chemicals that may have biological effects. In this respect, it is interesting that it is the amounts of a caffeine metabolite, the obrominel, rather than caffeine levels in the CSF that correlate with amyloid/tau markers in demented patients (Travassos et al., 2015). Interestingly, the intake of chocolate, rich in the obromine, is inversely correlated with memory deterioration (Moreira et al., 2016).

It is important to remarque that the neuroprotective effects of caffeine, associated to A_2A_ adenosine receptor inhibition, have been observed also in Parkinson’s disease, where much work has been carried out to demonstrate safety of the first A_2A_ adenosine receptor antagonist, istradefylline, recently launched as a new drug for this pathology in Japan (Nouriast) and in the United States (Nourianz) (Borea et al., 2016, Borea et al., 2017; Chen and Cunha, 2020). Istradefylline has been also shown to exert protective effects by reducing memory dysfunction in animal models of AD and for the future it would be crucial to determine whether it could also induce memory improvement in patients with AD (Orr et al., 2018). However, it should be underlined that istradefylline has a narrow therapeutic window in aging and experimental models of AD and PD, leading to the hypothesis that age and other factors may affect safety of A_2A_ receptor antagonists.

Finally, this opinion article has presented the main findings supporting the role of A_2A_ adenosine receptor antagonists on AD. Even though further work is necessary to better elucidate the mechanisms involved in the shift of A_2A_ receptor from beneficial target in normal synapses to detrimental one in aging and disease, its capability to modulate synaptotoxicity, glutamate-dependent NMDA signaling and calcium dysfunction, together with its effect on neuroinflammation, suggest a crucial role for its antagonism to prevent AD pathology.
